# Complete genome sequence of *Bacillus altitudinis* NBTC-002, isolated from agricultural ecological ditches

**DOI:** 10.1128/mra.00493-25

**Published:** 2025-09-10

**Authors:** Yueying Wang, Ling Chen, Wei Fang, Kaimei Wang, Lei Zhu

**Affiliations:** 1 National Biopesticide Engineering Technology Research Centre, Hubei Biopesticide Engineering Research Centre, Hubei Academy of Agricultural Sciences 117996, Wuhan, P. R. China; California State University San Marcos, San Marcos, California, USA

**Keywords:** ecological ditches, *Bacillus altitudinis*, complete genome

## Abstract

We presented the complete genome of *Bacillus altitudinis* NBTC-002 isolated from soil samples from ecological ditches on farmland, of which the total length is 3,799,862 bp and possesses 3,817 protein-coding sequences (CDS).

## ANNOUNCEMENT

Ecological ditches are artificial channels with planted aquatic plants, which can effectively intercept pollutants in agricultural drainage; however, the overall impact on agricultural nutrient removal remains insufficiently characterized (1). *Bacillus altitudinis* has been found to have potential for plant growth promotion (2) and bioremediation (3, 4). Strain NBTC-002 was isolated from 0–20-cm depth soil samples collected in October 2023 from farmland ecological ditches in Xiantao, Hubei Province, China (113.4430°N, 30.3278°E).

One gram soil samples were serially diluted with sterile water and were spread on lysogeny broth (LB) medium plates. After incubation overnight at 30°C, single colonies were purified via three rounds of scribing on LB plates. A purified colony was inoculated into LB medium and cultivated at 30°C to the middle logarithmic growth stage. Genomic DNA was prepared using the Blood & Cell Culture DNA Mini Kit (Cat#13323, QIAGEN, Germany) following the manufacturer’s instructions. DNA purity was measured using a NanoDrop One UV-Vis spectrophotometer (Thermo Fisher Scientific, USA). DNA samples were quantified using a Qubit 3.0 Fluorometer (Invitrogen, USA). The long- and short-read sequencings were performed from the same DNA preparation. For long-read sequencing, size-selected long DNA fragments (>10 kb) were then extracted using the PippinHT system (Sage Science, USA). The DNA library was prepared using a Ligation Sequencing Kit V14 (Cat#SQK-LSK-114; Oxford Nanopore Technologies, Ltd. [ONT], Oxford, UK) without DNA shearing and was sequenced with a Nanopore PromethION sequencer (ONT) on a R10.4.1 flow cell (FLO-MIN114). Base-calling and barcode segmentation, adapter sequence removal for the raw sequences were performed using Guppy v.5.0.16 (5). A total of 100,050 reads (1,562  Mb) were generated with a reads mean length of 15,616.99 bp, and N50 was 29,575 bp, and two reads were filtered out after base-calling and adapter trimming. For short-read sequencing, genomic DNA was randomly fragmented by Covaris LE220 focused ultrasonicator (USA), and the fragmented DNA was selected by MGIEasy DNA Clean Beads (Cat#1000005279, MGI Tech Co., Ltd., China) to an average size of 200–400 bp. A DNA library was prepared following the MGIEasy FS PCR Free DNA library preparation set (Cat#1000013455, MGI). Sequencing was performed on a DNBSEQ-T7RS platform (MGI), using paired-end 150 bp reads. Raw data (9,006,326 short reads with a total length of 1,350,948,900  bp) were processed using the FASTQ preprocessing program fastp v.0.23.1 (6) to trim adapters and low-quality data, yielding 8,804,784 clean reads (1,317,782,670  bp in total).

The complete genome of strain NBTC-002 (3,799,862 bp length in total) was assembled into two circular replicons through Unicycler v.0.5.0 (7): a 3,794,544 bp circular chromosome (41.53% GC content) and a 5,318 bp circular plasmid (35.97% GC content), with respective sequencing depths of 400.48× and 269.40×. Phylogenetic analysis using the Type (Strain) Genome Server (8) revealed a close evolutionary relationship with *B. altitudinis* type strain DSM 21631 (NCBI assembly accession no. GCA_000691145), supported by a digital DNA-DNA hybridization value of 86.4% calculated using GGDC formula 2. Genome annotation identified 3,817 protein-coding sequences (CDS), 81 tRNA genes, and 24 rRNA genes through NCBI PGAP v.6.10 (9). In this study, default parameters were used except where otherwise noted.

## Data Availability

The whole genome is available in GenBank under accession numbers CP187396 and CP187397. The raw sequencing data were deposited in the Sequence Read Archive (SRA) database under accession numbers SRR33097758 (ONT) and SRR33097759 (MGI).
